# Introducing W.A.T.E.R.S.: a Workflow for the Alignment, Taxonomy, and Ecology of Ribosomal Sequences

**DOI:** 10.1186/1471-2105-11-317

**Published:** 2010-06-12

**Authors:** Amber L Hartman, Sean Riddle, Timothy McPhillips, Bertram Ludäscher, Jonathan A Eisen

**Affiliations:** 1 Department of Medical Microbiology and Immunology and the Department of Evolution and Ecology, Genome Center, University of California Davis, One Shields Avenue, Davis, CA, 95616, USA; 2 Department of Computer Science, Genome Center, University of California Davis, One Shields Avenue, Davis, CA, 95616, USA; 3 Department of Biology, The Johns Hopkins University, 3400 N. Charles Street, Baltimore, MD, 21218, USA

## Abstract

**Background:**

For more than two decades microbiologists have used a highly conserved microbial gene as a phylogenetic marker for bacteria and archaea. The small-subunit ribosomal RNA gene, also known as 16 S rRNA, is encoded by ribosomal DNA, 16 S rDNA, and has provided a powerful comparative tool to microbial ecologists. Over time, the microbial ecology field has matured from small-scale studies in a select number of environments to massive collections of sequence data that are paired with dozens of corresponding collection variables. As the complexity of data and tool sets have grown, the need for flexible automation and maintenance of the core processes of 16 S rDNA sequence analysis has increased correspondingly.

**Results:**

We present WATERS, an integrated approach for 16 S rDNA analysis that bundles a suite of publicly available 16 S rDNA analysis software tools into a single software package. The "toolkit" includes sequence alignment, chimera removal, OTU determination, taxonomy assignment, phylogentic tree construction as well as a host of ecological analysis and visualization tools. WATERS employs a flexible, collection-oriented 'workflow' approach using the open-source Kepler system as a platform.

**Conclusions:**

By packaging available software tools into a single automated workflow, WATERS simplifies 16 S rDNA analyses, especially for those without specialized bioinformatics, programming expertise. In addition, WATERS, like some of the newer comprehensive rRNA analysis tools, allows researchers to minimize the time dedicated to carrying out tedious informatics steps and to focus their attention instead on the biological interpretation of the results. One advantage of WATERS over other comprehensive tools is that the use of the Kepler workflow system facilitates result interpretation and reproducibility via a data provenance sub-system. Furthermore, new "actors" can be added to the workflow as desired and we see WATERS as an initial seed for a sizeable and growing repository of interoperable, easy-to-combine tools for asking increasingly complex microbial ecology questions.

## Background

### Microbial communities and how they are surveyed

Microbial communities abound in nature and are crucial for the success and diversity of ecosystems. There is no end in sight to the number of biological questions that can be asked about microbial diversity on earth. From animal and human guts to open ocean surfaces and deep sea hydrothermal vents, to anaerobic mud swamps or boiling thermal pools, to the tops of the rainforest canopy and the frozen Antarctic tundra, the composition of microbial communities is a source of natural history, intellectual curiosity, and reservoir of environmental health [1]. Microbial communities are also mediators of insight into global warming processes [2,3], agricultural success [4], pathogenicity [5,6], and even human obesity [7,8].

In the mid-1980 s, researchers began to sequence ribosomal RNAs from environmental samples in order to characterize the types of microbes present in those samples, (e.g., [9,10]). This general approach was revolutionized by the invention of the polymerase chain reaction (PCR), which made it relatively easy to clone and then sequence rDNA (the genes for ribosomal RNA) in particular those for small-subunit ribosomal RNA (ss-rRNA). These studies revealed a large amount of previously undetected microbial diversity [1,11-13]. Researchers focused on the small subunit rRNA gene not only because of the ease with which it can be PCR amplified, but also because it has variable and highly conserved regions, it is thought to be universally distributed among all living organisms, and it is useful for inferring phylogenetic relationships [14,15]. Since then, "cultivation-independent technologies" have brought a revolution to the field of microbiology by allowing scientists to study a wide and complex amount of diversity in many different habitats and environments [16-18]. The general premise of these methods remains relatively unchanged from the initial experiments two decades ago and relies on straightforward molecular biology techniques and bioinformatics tools from ecology, evolutionary biology and DNA sequencing projects.

Briefly, the lab work involved in 16 S rDNA surveys begins with environmental samples (e.g., soil or water) from which total genomic DNA is extracted. Next, the 16 S rDNA is PCR-amplified with pan-bacterial or pan-archaeal primers (i.e., primers designed to amplify as many known bacteria or archaea as possible), cloned into a sequencing vector, and then sequenced (or directly sequenced without cloning in next generation sequencing) resulting in large collections of diverse microbial 16 S rDNA sequences from these different samples. As sequencing costs have continually declined, environmental microbiology surveys have expanded correspondingly and 16 S rDNA datasets have grown increasingly complex.

The size and complexity of data sets introduce a new challenge - analyses that one could carry out manually on small data sets now must be aided or run entirely on computers. And those analyses that previously were carried out computationally now must be made more efficient to have any hopes of being completed in a timely manner [7,19].

**Figure 1:**
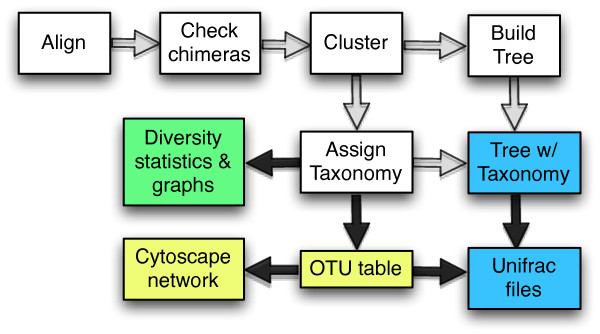
**Overview of WATERS**. Schema of WATERS where white boxes indicate "behind the scenes" analyses that are performed in WATERS. Quality control files are generated for white boxes, but not otherwise routinely analyzed. Black arrows indicate that metadata (e.g., sample type) has been overlaid on the data for downstream interpretation. Colored boxes indicate different types of results files that are generated for the user for further use and biological interpretation. Colors indicate different types of WATERS actors from Fig. 2 which were used: green, Diversity metrics, WriteGraphCoordinates, Diversity graphs; blue, Taxonomy, BuildTree, Rename Trees, Save Trees; CreateUnifrac; yellow, CreateOtuTable, CreateCytoscape, CreateOTUFile; white, remaining unnamed actors.

How then is the microbial community sequencing data converted from reads off a sequencing machine to bar graphs, network diagrams, and biological conclusions? Fortunately, even as data sets have expanded, most researchers analyzing rDNA sequence data sets, even when they are very large, have a similar set of goals in their analysis. For example, most studies are interested in assigning a microbial identity to the 16 S rDNA sequences and determining the proportion of these organisms in each sequence collection. And to achieve these (and related goals), a similar set of steps are used (Fig. 1) including aligning the rDNA sequences in a dataset to each other so that they are comparable, removing chimeric sequences generated during PCR identifying closely related sets of sequences (also known as operational taxonomic units or OTUs), removing redundant sequences above a certain percent identity cutoff, assigning putative taxonomic identifiers to each sequence or representative of a group, inferring a phylogenetic tree of the sequences, and comparing the phylogenetic structure of different samples to each other and to the larger bacterial or archaeal tree of life.

Over the last few years, a large number of software tools and web applications have become available to carry out each of the above steps (e.g., [20,21] for chimera checking, [22] for phylogenetic comparisons, STAP for taxonomy assignments). In practice, even as new software became available, researchers still have to act as the drivers of the workflow. At each step in this process, different types of software must be chosen and employed, each with distinct data formatting requirements, invocation methods, and each associated with a variety of post-analysis steps that may be selected and applied. Even after all of these steps have been completed, a wide variety of statistical and visualization tools are applied to these results to interpret and represent these data. In this context, there is a clear need for tools that will run a comprehensive set of analyses all linked together into one system. Very recently, two such systems have been released - mothur and QIIME. WATERS is our effort in this regard with some key differences compared to mothur and QIIME.

### Motivations

**Figure 2:**
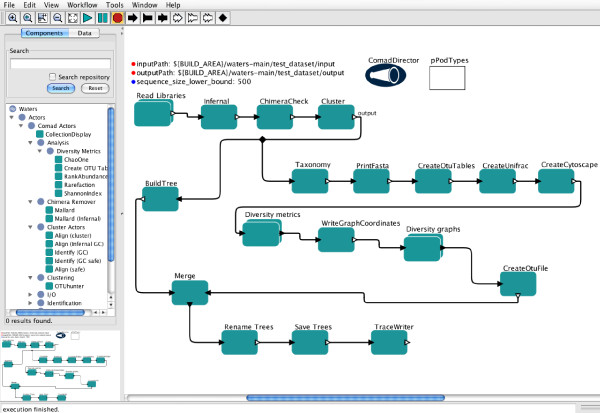
**Screenshot of WATERS in Kepler software**. Key features: the library of actors un-collapsed and displayed on the left-hand side, the input and output paths where the user declares the location of their input files and desired location for the results files. Each green box is an individual Kepler actor that performs a single action on the data stream. The connectors (black arrows) direct and hook up the actors in a defined sequence. Double-clicking on any actor or connector allows it to be manipulated and re-arranged.

As outlined above, successfully processing microbial sequence collections is far from trivial. Each step is complex and usually requires significant bioinformatics expertise and time investment prior to the biological interpretation. In order to both increase efficiency and ensure that all best-practice tools are easily usable, we sought to create an "all-inclusive" method for performing all of these bioinformatics steps together in one package. To this end, we have built an automated, user-friendly, workflow-based system called WATERS: a Workflow for the Alignment, Taxonomy, and Ecology of Ribosomal Sequences (Fig. 1). In addition to being automated and simple to use, because WATERS is executed in the Kepler scientific workflow system (Fig. 2) it also has the advantage that it keeps track of the *data lineage *and *provenance *of data products [23,24].

#### Automation

The primary motivation in building WATERS was to minimize the technical, bioinformatics challenges that arise when performing DNA sequence clustering, phylogenetic tree, and statistical analyses by automating the 16 S rDNA analysis workflow. We also hoped to exploit additional features that workflow-based approaches entail, such as optimized execution and data lineage tracking and browsing [23,25-27]. In the earlier days of 16 S rDNA analysis, simply knowing which microbes were present and whether they were biologically novel was a noteworthy achievement. It was reasonable and expected, therefore, to invest a large amount of time and effort to get to that list of microbes. But now that current efforts are significantly more advanced and often require comparison of dozens of factors and variables with datasets of thousands of sequences, it is not practically feasible to process these large collections "by hand", and hugely inefficient if instead automated methods can be successfully employed.

#### Broadening the user base

A second motivation and perspective is that by minimizing the technical difficulty of 16 S rDNA analysis through the use of WATERS, we aim to make the analysis of these datasets more widely available and allow individuals with little or no programming and bioinformatics skills to still use the best software currently available. Prior to WATERS, few microbiologists had the skills and time to invest for installing close to a dozen different pieces of software, troubleshooting them, preparing files, etc. if they wanted to do comprehensive 16 S rDNA analyses. With WATERS we believe more biologists can get to the heart of microbial ecology questions and obtain results faster.

#### Comparability and reproducibility

The third, complementary motivation to build WATERS was to "standardize" the 16 S rDNA analysis methods thus facilitating comparability and reproducibility of results. Although isolated reports have called for community-wide standardization of part of the 16 S rDNA analysis process [28], in the past most microbial ecologists have cobbled together software tools from different websites and individual software downloads in an ad-hoc manner that is hard to compare to other microbial analyses in other publications. In short, we sought to develop a reproducible, convenient "one stop shop" method for 16 S rDNA analysis that was accessible for a user with only minimal computational expertise. Data lineage and provenance information that is automatically generated during workflow runs provides rich additional opportunities for result validation and reproducibility [23-27]. Very recently, two new programs, Mothur [29] and QIIME [30], have been published that also attempt to standardize 16 S rDNA analyses. The similarities and unique attributes are discussed below and in Table 1.

**Table 1:** Comparison of WATERS' tools to existing web services and stand-alone software tools.

	Greengenes	RDP II	RDP-Py	Silva	Mothur	QIIME	WATERS
**Use**	Web	Web	Web	Web	Command line	Command line	GUI
**Align**	NAST	Infernal	Infernal	SINA	NAST		Infernal
**Chimeras**	Bellerohpon	No	No	No	Unknown		Mallard
**OTUs**	Yes	DOTUR	Complete-linkage	No	DOTUR		OTUHunter
**Taxonomy**	Simrank; 7mer classification	naïve Bayesian classifier	naïve Bayesian classifier	Yes	Yes		STAP
**Trees**	No	NJ	NJ	No	Yes		ML; NJ
**Ecology**	No	No	Yes	No	Yes		Yes
**Unifrac**	No	No	No	No	Yes		Yes
**Export**	Yes	Yes	No	Yes	No		No
**Trim?**	Yes	Yes	Yes	No	Yes		No
**Data size?**	hundreds	hundreds	500,000	hundreds	tens of thousands	tens of thousands	tens of thousands

Along the left column, "Use" indicates where or how the software is used; "Align" indicates the alignment programs available; "Chimeras" indicates the chimera removal software available; "OTUs" indicates the software used to detect and determine operational taxonomic units; "Taxonomy" indicates the software used to assign taxonomy to OTUs; "Trees" indicates the software used to build phylogenetic trees; "Ecology" indicates whether or not ecological indices such as Chao1 and the Shannon index are calculated; "Unifrac" indicates whether Unifrac analyses are done within the software or whether data is formatted for downstream use in Unifrac; "Export DB" indicates whether a quality-controlled, curated 16 S dataset is available for export and/or for comparison to the user's own dataset; "Trim" indicates the availability of quality control trimming to remove sequence vectors or low-quality bases from the initial upload of sequences; "Dataset size" indicates the estimated amount of sequences that can be readily processed through each software type. Along the top are all known multi-tool 16 S rDNA analysis software suites. Note that these software are each under very active development. This table represents a snapshot in time of current tool availabilities. ML, maximum-likelihood; NJ, neighbor-joining.

### Scientific workflows and the Kepler system

In recent years, the concept of automating repetitive, complex informatics tasks has gained popularity and practice in many scientific communities [25,31], and been widely used and implemented in the public sector for corporate use. This process, when applied to scientific research, is termed *scientific workflow automation *[31], and a variety of different scientific workflow systems are available or under active development (e.g., [31,32]). Based on our prior experience extending the Kepler scientific workflow system, we chose to implement WATERS in Kepler [33]. The use of a scientific workflow system in general and Kepler in particular offers several advantages for use by the scientific community [25], which are described below.

First, it is open-source and freely available, and thus ideal for academic development. Second, unlike other systems, Kepler is independently extensible, which means that developers can make changes to the underlying workflow system if the need arises. To our knowledge, no other workflow system allows developers to make major changes to the system for their particular application needs. For example, as part of WATERS, a custom data cache capability was developed that allows for incremental recomputation of results (see below). Such enhancements can also be contributed back to the shared source code repository and used by other projects.

Kepler is also unique in that it supports different models of computation (e.g. different forms of executing dataflow process networks, streaming pipeline parallelism, etc.) via software components called *directors*. For example, WATERS employs a new COMAD director (Fig. 2) to simplify handling of nested data collections [23,25,27]. Similarly, developers who need to make deep changes to the system, e.g., in order to change workflow scheduling or data handling for specific applications or projects, can do so by customizing existing directors or devising new ones. Other contemporary workflow systems do not allow such customization and instead only support a single way of running workflows (deep changes to these systems would likely break existing workflows).

Fourth, a very active Kepler community is constantly developing actors, the individual units within a workflow that perform specific operations on the data, for many fields in the natural sciences [34]. These actors now make up a large library, available via a public repository of usable, interoperable, and interchangeable actors.

Kepler has been used in a wide variety of scientific domains and communities, ranging from astrophysics, to ecology, to particle physics [31], and - importantly for 16 S rDNA analyses - the phylogenetic and ecology communities [26], which have similar needs and functions. In fact, the RAxML actor that builds phylogenetic trees within WATERS already existed prior to the development of our workflow, demonstrating the intrinsic reusability and exchangeability of workflow actors.

#### Incremental Recomputation

Finally, Kepler has a built-in database that allows calculations to be cached and stored internally rather than recalculated anew every time. For instance, in analysis of 16 S rDNA datasets, new data often become available sporadically as sequencing centers complete batch jobs. The addition of new data generally requires re-analysis of the entire dataset, but, by using the cache, previous intermediate data products, including alignments, chimeras, and taxonomy assignments, can be retrieved automatically from the database rather than being recalculated. Therefore, the cache increases the efficiency of adding new data to a partially analyzed dataset. Moreover, if new metadata parameters become available or are altered, the entire workflow can be re-run on the existing cached data and all new results files can be generated without the need for any heavy recalculations.

## Implementation

Scientific workflow systems typically represent workflows as networks of components (representing workflow steps, tasks, or processes). In Kepler, these components are called *actors *which can be viewed as independently executing processes, and which communicate by sending data through unidirectional pipelines (a.k.a. *channels*). New workflow components can be added simply by choosing new instances of existing actors, or by building new "native" actors, i.e., implemented in Java, the underlying implementation language of Kepler. A workflow consisting of actors and their dataflow connections is then executed according to a schedule as prescribed by the director (see Fig. 2).

Kepler supports a number of ways to add new actors to the system and to implement new actors. If the desired actor is not available from the library or a remote actor repository, one can either create new "native" Java actors, or one can instantiate certain generic actors in new ways. The former requires programming expertise in Java, while the latter doesn't. For example, to add a new data analysis step implemented in the R language as an actor, one only has to instantiate the generic R actor accordingly. This specialized R actor instance can then be stored as a new actor. Other ways to add new actors to Kepler include, e.g., instantiation of the command-line actor (effectively "wrapping" a given command-line tool and turning it into an actor), or instantiation of the web service actor so that certain web services become new components (actors).

For WATERS a number of custom actors were developed to perform the required microbial ecology functions. Some, like the Mallard and OTUHunter actors (described below in detail), directly invoke pre-existing Java-based algorithms. Others, like the STAP and Infernal actors, invoke external non-Java programs automatically, meaning that these actors run the programs on behalf of the WATERS user, wait for the results to be produced, and reincorporate these results into the internal data stream seamlessly. Two additional features of WATERS are the options to use a computer cluster to accelerate compute-intensive processes (the Infernal, OTUHunter, and STAP actors all can take advantage of a cluster) and to intelligently reuse existing results within the cache where possible to minimize computation.

The main WATERS workflow comprises 23 actors, each of which is a Java class that implements a common actor interface. This interface allows the workflow system to communicate with the actor, directing it to take certain actions. The COMAD (Collection-Oriented Modeling and Design) computational model within Kepler was used [27]. In the COMAD style of developing and executing workflows, a stream of structured data flows through a sequence of actors that together form a computational pipeline. Much like workers in an assembly line, each actor in the pipeline can add or remove information from the data stream passing through it; newly computed data products are added to the stream, and data that will not be needed by downstream actors can be deleted. Actors that conform to the COMAD paradigm need not process all the data that streams through them; instead, the workflow designer can declare with an XPath-like syntax, (XPath is a simple query language for selecting nodes from an XML data stream) which data in the stream each actors should process, giving the workflow designer many options for composing the structure of the data stream, grouping intermediate and final results with the raw data used to compute them, etc. It has been shown that this workflow modeling paradigm results in more robust, flexible, and change-resilient workflows when compared with conventional workflow designs [25,35].

### The WATERS Workflow

In this section we describe step-by-step the analysis automated by the WATERS workflow and portrayed in Fig. 1.

#### Sequence libraries import

Because most sequencing centers in our experience return to users assembled quality-controlled contigs, the workflow begins its operation by importing a collection of sequences in FASTA format. The design only assumes that the user has a collection of libraries generated from individual samples (neither the size of the libraries nor the number of samples is fixed). The only other information input to WATERS is a file that allows the user to assign a single metadata table to the input libraries. Each line of this file corresponds to one library, and any number of columns corresponding to distinct variables may be included in the table and used to group data during downstream analysis. This metadata is used, for example, to indicate from which environment or experimental condition each library was generated.

#### Sequence alignment

For the first step, sequence alignment, two options are available. The default is the recently released Infernal package [36]. Infernal has the advantage over previous alignment methods that it takes into account the secondary structure of the 16 S rRNA molecule, and that it is extremely fast. Because it is able to discern homologous positions of secondary structure it can more easily and accurately determine group-specific insertions, which it subsequently removes from the alignment. Infernal also very efficiently performs its alignment one sequence at a time, and thus can take advantage of parallel processing capabilities, e.g., of Linux clusters.

Alternatively, the STAP aligner, which uses the ClustalW algorithm for alignment and is part of the STAP package for taxonomy assignment [37], may be employed. While the STAP aligner does not take into account rRNA secondary structure, it also is fast and is the same alignment method used during the downstream taxonomy assignment step (see below). Additionally, many short, bar-coded (454) sequences are from the variable regions of the 16 S molecule, which Infernal is unable to align. Providing this alternate aligner gives the user flexibility depending on the type of sequences they are using and allows for comparisons between the two approaches.

#### Chimera removal

During the PCR amplification process two non-identical single-stranded pieces of DNA occasionally will anneal together at regions of high sequence identity. DNA polymerase can amplify such hybrid pieces of DNA because one piece of single-stranded DNA can serve as a primer for DNA replication and thus will lead to chimeras when the "primer" portion is of separate origin from the part added downstream of the primer. This will essentially contaminate the PCR product with chimeric sequences that were not present in the original sample. Because these artificial sequences can skew the interpretation of the real data, they should be (computationally) removed from the input sequence libraries before further analysis. However, bioinformatics tools for detecting chimeras are still relatively new, and only a few programs are available to choose from. The Mallard program [20], based on the Pintail algorithm [38], is widely-used for chimera removal and was selected for use in WATERS. However, to automate Mallard from within WATERS its graphical user interface (GUI) had to be eliminated for automation of the algorithm, although the program's parameters are configurable within the actor (through double-clicking on it) and all of the original confidence interval options are available. An additional Chimera checking program, Bellerophon, was considered because it employs a different chimera detection methodology [21] and could theoretically complement Mallard. However, Bellerophon is not yet available as a stand-alone package. Chimera detection algorithms continue to be optimized and improved, and, in time, perhaps the chimera-removing actor in WATERS can be upgraded and replaced as newer software becomes available.

#### Determination of OTUs

A key step in determining the total number of times a particular microbe is represented in a library is the clustering of highly similar sequences together to infer operational taxonomic units (OTUs). OTUs are akin to molecular microbial species and are commonly based on 97% or 99% sequence identity levels. A single representative sequence is chosen from each cluster, and the count of sequences in the cluster can be used to compute the relative proportion of the corresponding OTU in the total collection of sequences. Those sequences not chosen to represent the OTU in which they were clustered may be excluded from further analysis because their presence is included in the total sequence count for the OTU.

A new piece of software, OTUHunter (Huntemann, et al, in preparation), is used for clustering sequences and identifying OTUs in WATERS. OTUHunter uses an implementation of the Markov clustering (MCL) algorithm to group sequences together (Huntemann et al, in preparation). Briefly, OTUHunter first calculates an all-by-all similarity matrix (using the Kimura 2-Parameter) from the alignment of the sequences in all libraries. It then simulates random walks via the MCL algorithm on the graph represented by the similarity matrix and measures the flow until the algorithm ends in a nearly idempotent matrix (meaning the flow has stabilized and change has ceased). The resulting matrix is interpreted as the clusters, and the sequence in a cluster over which the most flow went is chosen as a representative of that cluster (termed the "OTU representative" in the resulting output text file). If there is more than one sequence with the same amount of flow within a cluster, one of them will be chosen arbitrarily as the representative.

#### Taxonomy assignment

Taxonomy assignments are made for each of the representative sequences chosen by OTUHunter. The STAP software [37] is used for taxonomy assignments and can either be run locally or be configured to take advantage of the parallel processing capabilities of a Linux cluster. As a side effect of our development of WATERS, STAP can now be used in WATERS alone without pre-compilation by simply deleting all other actors in the workflow and creating a very short workflow involving the FASTA input and STAP actors. Prior to WATERS, STAP only could be run from the command line and after going through several installation steps.

#### Phylogenetic tree inference

WATERS infers phylogenetic trees relating the OTU representatives using either the neighbor-joining-based programs, Fasttree [39] or Quicktree [40], or the maximum-likelihood program, RAxML [41]. A RAxML actor had been developed previously and released as part of the Kepler/pPOD package [26]; it submits compute jobs to the CIPRES (**C**yberinfrastructure for Phylogenetic **Res**earch: http://www.phylo.org) cluster at the San Diego Supercomputer Center [42]. We also developed new Kepler actors to run Fasttree and Quicktree as part of WATERS.

### Workflow development and testing

WATERS was created in an iterative, bottom-up manner, starting from small pieces of the overall target workflow. This iterative development allowed easy adaptation while the user requirements were still evolving. Initial testing was performed by using small collections of sequences as test datasets and sequentially adding new actors to the workflow, identifying and solving error messages produced, and refining the workflow and Java code until the final version was produced. First, two test users within the lab (of JAE, seek Acknowledgements) with different programming and bioinformatics skills were employed to use the workflow and provide feedback on WATERS setup and usage. Next, three test users outside of UC Davis were asked to experiment with WATERS, report any bugs or problems encountered, and comment on the user manual. WATERS was also tested with increasingly large datasets to detect any scaling issues. Testing revealed certain performance bottlenecks, both in parallelization and in memory usage, which were subsequently removed and optimized.

## Results and Discussion

After downloading and running WATERS, a variety of results files are automatically generated (Table 2) and are available for biological interpretation and comparison. These files and their use are discussed below. Next, a discussion is presented of how the workflow can be customized and optimized for specific requirements and advanced usages. Then, we discuss other types of 16 S rDNA tool suites currently available and discuss specifically where WATERS fits. Finally, the broad benefits of automation and its implications for the community are discussed and some of the limitations of the WATERS method are acknowledged.

**Table 2:** Results files generated by WATERS for further analyses.

Results file	Contents	Purpose
aligned_sequences.fas	All seqs pre-OTUHunter	QC; Alignment
bad_infernal_sequences.fas	Seqs un-alignable by Infernal	QC
chimeras.fas	Seqs removed by Mallard	QC
coordinates_Rank_abundance.csv	x,y coordinates for rank-abundance	Create graphs
coordinates_Rarefaction.csv	x,y coordinated for rarefaction	Create graphs
graph_*_variable.xgmml	Similarities between libraries based on shared OTUs	Cytoscape
graph-Rank_Abundance.bip/.ps	Printed graph of rank-abundance curves	View graphs
graph-Rarefaction.bip/.ps	Printed graph of rarefaction curves	View graphs
otu-table.txt	Counts of OTUs and diversity indices at each cutoff and metadata variable	Graph OTUs; diversity metrics
sequences-*.fas	One representative seq for each OTU found	Alignment
short_sequences.fas	Short seqs that did not pass cut off	QC
tree_*.txt	Phylogenetic tree of representatives with taxonomy information	Unifrac Dendroscope
unifrac_*_variable.txt	"Environment file" for Unifrac; OTU abundance and library info	Unifrac
workflow.trace	Provenance file written by Kepler describing the worklow run	QC

Fourteen different types of results files can be generated from one run of WATERS in its complete configuration. * represents the cutoffs used in OTUHunter, by default 97 and 99 percent similarity, which will generate two different files at each cutoff used. Abbreviations: Seq(s), sequence(s); QC, Quality Control; OTU, Operational Taxonomic Unit.

### Results files automatically generated and delivered to the user

#### Ecology statistics

One of the first questions a microbiologist may likely have about their community of interest is how many kinds of organisms do I have here? And how different is one sample from another? To answer these questions two diversity indices are calculated: Chao1 and the Shannon index. The Chao1 diversity index [43] is an ecological statistic that estimates the total number of species in a collection by taking into account the total size of the sample and the number of times a sequence was seen at least twice. This is a measure of "richness", i.e., total estimated number of organisms. A Shannon-Weiner index [44] is also calculated, which is a measure of "evenness" that includes a measure of richness but also takes into account the relative proportion of the organisms in the collection, to convey how evenly distributed the organisms are throughout the sample.

**Figure 3:**
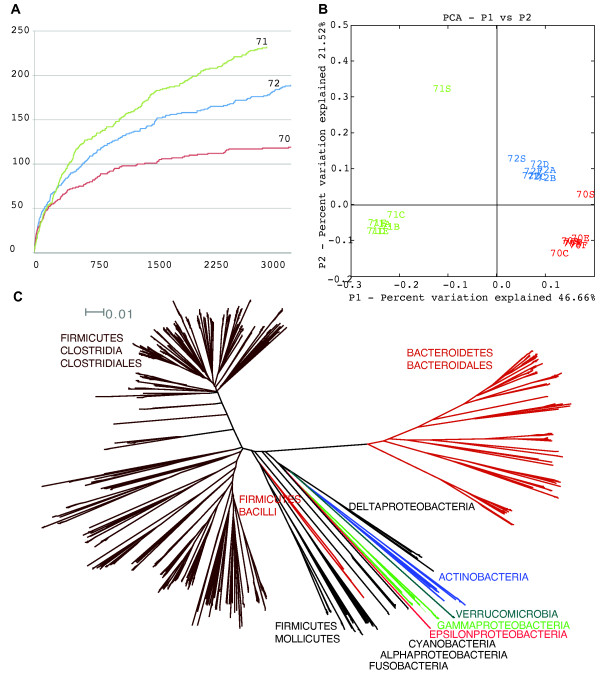
**Biologically similar results automatically produced by WATERS on published colonic microbiota samples**. (A) Rarefaction curves similar to curves shown in Eckburg et al. Fig. 2; 70-72, indicate patient numbers, i.e., 3 different individuals. (B) Weighted Unifrac analysis based on phylogenetic tree and OTU data produced by WATERS very similar to Eckburg et al. Fig. 3B. (C) Neighbor-joining phylogenetic tree (Quicktree) representing the sequences analyzed by WATERS, which is clearly similar to Fig. S1 in Eckburg et al.

Two other global ecological results are returned: the calculation and display of rarefaction curves (Fig. 3A) and rank-abundance curves. In WATERS these curves are displayed as a graphical pop-up image, a saved post-script image file, and a text file that contains the x, y coordinates of the graphs. The rarefaction curve displays the increase in the number of OTUs as more sequences are added to the collection. The slope of the rarefaction curve indicates how well sampled a library or an environmental (metadata) variable was. For example, as the rarefaction curve begins to flatten out (asymptote) along the x-axis, very few new OTUs are being added as new sequences are added (Fig. 3A). The rank-abundance curve plots along the x-axis, in decreasing order, the most abundant organisms in a sample and presents on the y-axis the quantity of that organism, i.e., organism rank is displayed on the x-axis and abundance on the y-axis, therefore, presenting an overview of the distribution of organisms in a sample.

#### Library comparisons

One useful file produced by WATERS is the OTU table (Table 2). The OTU table writes out the OTU representatives, taxonomic description of these sequences and the total size of an OTU on a per library and per metadata variable basis (at all chosen cutoffs of OTUHunter). The information contained in this file can be employed in many practical ways to get an overall picture of how libraries compare. For example, by dividing the abundance of each OTU by the total number of high-quality reads in a library a relative proportion of each OTU can be determined. These relative OTU abundances can then be imported into heat-mapping software, e.g., TreeView [45] microarray visualization software. The heat map can then be clustered based on relative abundance and then on similarities between libraries in a manner similar to clustering gene expression patterns generated from microarray data. These relative or raw OTU values or even a presence/absence view of the OTUs (simply made in Excel by changing all numbers greater than 0 to a 1) are also formatted for use with many different statistical tests such as PCA or hierarchical clustering. Additionally, the OTU table can be used to sum up taxonomy groups at any taxonomic level to produce taxonomy bar graphs.

Two additional sets of results (Table 2) are produced which are pre-formatted for use in specific software programs that allow the user to globally compare libraries. First, are the files required for phylogenetic library comparisons in Unifrac [22], namely a phylogenetic tree and an "environment" text file. The Unifrac program allows the user to determine the statistical over- or under-representation of microbial lineages based upon their phylogeny, i.e., the **Un**ique **fra**ction of the phylogenetic tree branch length present. Second, files are formatted for use in a network-viewing program, Cytoscape [46]. Cytoscape does not take into account phylogeny, but creates a clear visual image for how similar two groups of samples are based upon the shared number of OTUs. For both Unifrac and Cytoscape files, results are generated for every similarity cutoff used in OTUHunter (the default is 97% and 99%), and they are also generated for every metadata variable comparison that the user includes.

#### Data pruning

To assist in troubleshooting and quality control, WATERS returns to the user three fasta files of sequences that were removed at various steps in the workflow. A short_sequences.fas file is created that contains all sequences that were removed because they did not meet the length requirements (default is 500 bp). Mallard creates a chimeras.fas file that contains sequences that were determined to be likely chimeras and thus removed. And a bad_infernal.fas file is created that contains sequences that were unalignable by Infernal.

### Flexibility and adaptability

The basic WATERS workflow (file: full.xml) contains specific default settings, but can be significantly altered and adapted in a number of different ways: e.g., the basic workflow can be changed by adding, removing, or rearranging individual actors, by changing workflow and actor parameters, and by modifying the metadata file. For runs with large amounts of data, an external MySQL database can be used instead of the built-in Kepler database. The following sections discuss these possibilities and the user manual gives more specific detail on how to implement and select these changes.

#### 1. Workflow Design Changes

Many scientific workflows are, at least initially, exploratory in nature, i.e., unlike a production workflow that is used over and over again without changes to the design, exploratory workflows evolve, while the scientist is experimenting with the workflow design, testing outputs for different input datasets, parameter settings, methods used, etc. The visual programming paradigm of scientific workflows makes it fairly easy for scientists to add, remove, or substitute workflow components while exploring alternative workflow designs, in particular when a change-resilient workflow paradigm such as COMAD is used. Conventional workflow designs often require special adaptors (a.k.a. "shims") and rewiring of workflows when design changes are made or when the structure of data changes. In contrast, COMAD can adapt to these changes via corresponding changes to its actor signatures and configuration parameters (e.g., read-scope) [25,35].

Beyond simple deletion and addition of existing components, workflow actors can also be exchanged to provide different methods or implementations for conceptually similar workflow steps. For example, there are two types of alignment methods, STAP and Infernal, Infernal is the default method but can be removed and swapped out with the STAP aligner. Or, alternatively, the aligner can be disabled entirely if the user wishes to use their own alignment format, such as Greengenes [47], or has manually aligned the sequences. Additionally, the phylogenetic tree methods can be switched out between RaxML [42], Fasttree [39], and Quicktree [40].

#### 2. Workflow and Actor Parameters

The second category of flexibility is to change the parameters of the overall workflow or of specific actors. For example, Mallard, OTUHunter, and STAP each come with default parameters, but depending on the individual requirements of the user, the default settings can be changed by double-clicking on the actor and altering the parameters. Mallard can be made to be more or less stringent in detecting chimeras, OTUHunter can calculate OTUs at different percent identity cutoffs, and STAP can search for taxonomy information in bacterial, archaeal, prokaryotic or eukaryotic domains of life. Additionally, there is a default sequence length cutoff at the beginning of the workflow that can be changed from the default setting of 500 bp minimum.

#### 3. Deployment on a compute cluster

The compute-intensive actors of WATERS can also be executed on a linux cluster rather than locally on the scientist's desktop or laptop. Deploying the analyses from these actors allows the compute time to be increased because parallel computing can be taken advantage of. The cluster-deployable actors are (i) the STAP aligner, (ii) the STAP taxonomy assigner, (iii) OTUHunter, and (iv) Infernal. To turn on the cluster submission process, a radio button is checked and the user's account information for the cluster is entered if necessary for the user's cluster account. For extensive technical information on this process please see the online documentation wiki for more information: http://code.google.com/p/waters16s/wiki/ServerActors.

#### 4. Change metadata

Another way to produce new kinds of results is through the user-written metadata file. This metadata file is optional, but allows the user to describe the libraries analyzed in any combination of variables of interest. For example, many variables of interest can be added on the fly as the user becomes aware of additional experimental parameters or begins to test a new hypothesis as new variables of interest develop as a result of the original analysis. Each variable column is used to create new Unifrac, Cytsoscape and OTU table files/text. Notably, because of the caching and incremental computation feature in WATERS, unnecessary re-computations after metadata file updates can be avoided.

#### 5. Switching to a different database

Kepler has a built-in HSQL database that is well-suited for smaller sequence collections of up to approximately10,000 sequences. For analyzing larger datasets we have made WATERS compatible with a MySQL database which will make the resulting results cache from larger runs more robust, quick and stable.

### WATERS proof-of-principle results are biologically meaningful

To test WATERS we used data from a published dataset of ~11,000 full-length 16 S rDNA sequences from the human colon [48]. The total number of OTUs (akin to bacterial species) at the 99% similarity cutoff was comparable: 381 determined by WATERS vs. 395 as published. The OTUs were further analyzed (Table 3) and show that both the OTU abundance as well as the number of OTUs in each taxonomic group are very similar. In most taxonomic groups there is complete or near-complete numerical correspondence between the two datasets. The biggest discrepancy lies in the Firmicutes Clostridia group where a smaller quantity was observed in the WATERS results. The possible cause(s) of this difference are unknown. The biological results (Fig. 3A-C) were also highly concordant with previously published results. The rarefaction analysis (Fig. 3A) indicated that the overall picture of sequence diversity was similar to published results. The UniFrac analysis (Fig. 3B) indicated that the microbiota similarity was responsible for the clustering of samples together and for the separation of different individuals away from others. The topology of the phylogenetic tree (Fig. 3C) indicated that the diversity and distribution of the microbiota was also consistent with published results.

**Table 3:** Comparison of OTU abundance between WATERS' automated results and previously published data.

Taxonomy	WATERS		**Eckburg et al**.	
	Abundance	OTUs	Abundance	OTUs
Actinobacteria	18	10	22	10
Alphaproteobacteria	10	4	10	4
Bacteroides	5510	67	5640	65
Betaproteobacteria	27	6	32	5
Cyanobacteria	3	1	3	1
Deltaproteobacteria	24	4	24	4
Epsilonproteobacteria	2	1	2	1
Firmicutes Clostridia	4849	265	5721	274
Firmicutes Mollicutes	318	19	287	27
Fusobacteria	9	1	9	1
Gammaproteobacteria	5	2	5	2
Verrucomicrobia	69	1	76	1

Along the left are the bacterial taxonomic groups detected in the dataset. Across the top are the results from WATERS compared to the previously published results. Columns 1 and 3 provide the total abundance of all OTUs in that taxonomic category. Columns 2 and 4 provide the number of discreet OTUs observed in that taxonomic group. OTU abundance data for the Eckburg et al. dataset can be found on page 23 of the original publication's supplemental material [48].

WATERS was also used to analyze the nine small bowel transplant libraries described in Hartman et al. [49]. These sequence collections were used to optimize and develop WATERS at both a small scale [49] and large scale (Hartman et al., in preparation) of sequence collections. The data were analyzed using WATERS and the results described were derived from output files generated by WATERS. The results from these analyses were used to set the default parameters in WATERS.

### Advantages of using a scientific workflow approach

The automation of 16 S rRNA analysis through a workflow system offers several advantages. First and most obviously, WATERS dramatically increases the efficiency of these analyses. WATERS saves both human time and compute-time thus allowing scientists to focus on result analysis and biological interpretation rather than on repetitive and often error-prone manual data handling tasks. WATERS also provides new means for the validation, "debugging", and reproducibility of results: The workflow produces log and trace files that capture exactly what operations and calculations have been performed and how derived products were obtained from their inputs.

These provenance capabilities [23,24] provided by WATERS through Kepler effectively turn the system into an automatic lab notebook that records details that are normally not captured and published. With increasingly powerful means to browse and query provenance in Kepler [50] and similar systems such as Taverna and Vistrails [51], and with the emergence of standards for data provenance such as Open Provenance Model (OPM), it will only become easier in the future to interpret, validate and re-analyze workflow results, both for the original experimenters and for other users.

The use of a workflow approach also increases the size of a workable dataset, and, in parallel, should greatly decrease user-mediated error. Our own experience of performing large-scale analysis with self-written Perl scripts strung together with shell commands has taught us that unintentional bioinformatics errors can be quite common in practice and may be easily perpetuated throughout an entire dataset. By handing over the bookkeeping to Kepler and concentrating efforts on the results and interpretation, the impact of human error is minimized, particularly those borne out of minimal expertise or minimal software familiarity. Ultimately, this allows for larger, more complex analysis and comparisons, which should enhance the broader field of microbial ecology. Furthermore, because the analysis processes always occur in the same manner, WATERS could be very useful for comparing and contrasting different published datasets to each other.

Tying together software written in different programming languages, requiring different inputs, returning varied outputs, and expecting different types of user interactions into one workflow program is a large technical challenge. The technical considerations to building a robust system composed of these varied and different software parts were quite high. This is one of the first examples of a wide distribution of a fully self-contained Kepler workflow package (which includes executable versions of diverse pieces of software) and, to the best of our knowledge, is the first comprehensive and extensible 16 S rDNA analysis package of its kind.

### Known Limitations

The current WATERS release bundles a number of external software components and is initially available and supported on Mac OS × (Leopard). Ports to other platforms are planned for the future (Kepler itself is platform independent and is distributed on Windows, Mac OS, and Linux). Instructions for how to set up and use WATERS on other operating systems are available on the WATERS web site; however, they are currently not well supported or tested.

Some data and metadata entry is currently file-based, i.e., users have to conform to the specific file formats to ensure the workflow executes as expected. In the future, we plan to include a more convenient form of data and metadata entry, e.g., using a configuration wizard. This will also minimize execution errors due to formatting errors in the input files.

Similarly, deploying WATERS on a cluster requires the user to make sure the cluster-setup has been done correctly. Working with distributed computing resources and similar parallel computing middleware is inherently more complex than working with a single machine. The emergence of virtual computing environments and cloud computing services should make deployment of WATERS on parallel platforms much more easy and fault-tolerant in the future.

Using our machines and setup, we could run WATERS roughly around 50,000 full-length sequences. This limit is imposed by Java memory limits in Kepler and in OTUHunter clustering, but will vary somewhat depending on the similarity of sequences run; more similar sequences will lower the sequence limit because the clustering becomes more memory intensive. Despite these limitations, WATERS provides end users with a new, more intuitive and user-friendly approach than has currently been available for 16 S rDNA analysis.

### Related 16S rDNA analysis tools

Mothur [29]is a tool with similar aims as WATERS, but which uses a more conventional command-line approach instead of employing a scientific workflow system approach. The software tools it incorporates are different from those available in WATERS. Table 1 shows a comparison of Mothur, WATERS, and other tools at the time of writing:

Three large websites are specifically dedicated to the purpose of 16 S rDNA microbial ecology, Greengenes [47], RDP-II [52], and Silva [53]. Each website has many, but not all, of the tools required for a high quality community analysis. Additionally, they all depend on web services, which inherently limit autonomy and sometimes limit the speed and the amount of data that can be processed (Table 1). Furthermore, these websites currently do not return data in advanced formats to be used in programs like Unifrac [22], Cytoscape [46] or heat map visualization. They also do not report ecological calculations like diversity metrics, rarefaction analysis or rank-abundance curves.

Recently a new pipeline, email-based tool for short bar-coded 454 pyrosequencing datasets became available within RDP (unpublished, http://pyro.cme.msu.edu/). The idea and concept are also similar to WATERS, but the input data is limited to 454 reads (100-250 bp) rather than full-length reads as WATERS is designed for. It is included in the table for comparison purposes, but is not designed for the same type of data. Table 1 compares WATERS to the available 16 S rRNA gene analysis web services as well as stand-alone programs that serve similar functions.

In summary, WATERS provides the community with a comprehensive and flexible 16 S rDNA analysis platform in a single stand-alone software package. WATERS can be installed on the user's local machine, but also scales to very large datasets with its support for cluster deployment of several compute-intensive steps. The WATERS workflow can also be customized and evolved, according to the user's needs.

### Future directions

As new sequencing methods continue to be developed, with decreasing costs, WATERS can be easily adapted for the data delivered by those "next generation" sequencing technologies. Specifically, short bar-coded sequences of the variable regions from pyrosequencing require a great deal of pre-screening and quality control. In order to adapt WATERS to pyrosequencing reads, new actors that perform these functions need to be implemented. Furthermore, OTUHunter is currently the rate-limiting computation within the workflow and would either need to be optimized or replaced for datasets that exceeded roughly 50,000 sequences or more as pyrosequencing datasets would surely do.

Fortunately, though, besides additional screening and clustering, many of the downstream actors in WATERS would continue to be useful, and, in fact, if widely adapted, would allow for easier comparisons between methods or between datasets made with two different technologies. The beauty of the workflow system is modularity. WATERS grants an opportunity to expand, adapt, and change without disrupting the functional parts that already exist.

## Conclusions

We present here a new, automated, workflow system to analyze 16 S rDNA clone libraries. The system is flexible, evolvable, and modular; workflow results can be inspected and validated using the built-in data provenance sub-system, facilitating the reproducibility of results. WATERS should increase community-wide the transparency of results and data management as well as allow new, less-experienced users to perform analyses at a very high technical level that might otherwise be too overwhelming. WATERS increases efficiency and saves time; ultimately allowing the user to concentrate their efforts on more biologically-engaging questions about microbial communities rather than on repetitive bioinformatics tasks. In short, microbial ecologists do not need to be constrained by their programming abilities in order to ask penetrating comparative microbial ecology questions if they employ WATERS.

## Availability and requirements

**• Project name: **WATERS

**• Project home page: **waters.genomecenter.ucdavis.edu; user manual and problem-solving wiki also available here

**• Operating system: **Mac OS 10.5 and 10.6

**• Other requirements: **Java 5

**• License: **MIT open-source

WATERS can be downloaded from waters.genomecenter.ucdavis.edu, which re-directs to a Google Code project page. The download is large (~500 MB) due to the dependencies of the STAP application take up around 100 megabytes, and the self-contained installation of Perl and TCL add another 200 megabytes. The remainder is the source code and resources that make up Kepler.

On the site, a wiki contains several entries that explain how to use WATERS at several levels of complexity. The Issues section of the site allows users to report any bugs encountered to the developer (SR). Currently, the stand-alone version of WATERS (all programs bundled together in one simple package) is available for Mac OS × (10.5 and 10.6). Windows and Linux users will need to pre-install several programs before using WATERS and should follow the instructions on the wiki.

## List of abbreviations

WATERS: Workflow for Alignment, Taxonomy, and Ecology of Ribosomal Sequences; rDNA: ribosomal DNA

## Authors' contributions

ALH, JAE, and BL wrote the paper; ALH, SR, TM, BL, JAE designed research; SR programmed WATERS; ALH and SR tested WATERS. All authors read and approved the final manuscript.
